# A Mindfulness-Based Compassionate Living Training in a Heterogeneous Sample of Psychiatric Outpatients: a Feasibility Study

**DOI:** 10.1007/s12671-016-0518-8

**Published:** 2016-05-12

**Authors:** Agna A. Bartels-Velthuis, Maya J. Schroevers, Karen van der Ploeg, Frits Koster, Joke Fleer, Erik van den Brink

**Affiliations:** Center for Integrative Psychiatry, Lentis Mental Health Organization, Groningen, The Netherlands; University Center for Psychiatry, University Medical Center Groningen, University of Groningen, Groningen, The Netherlands; Department of Health Psychology, University Medical Center Groningen, University of Groningen, Groningen, The Netherlands

**Keywords:** Self-compassion, Mindfulness, Depression, Anxiety

## Abstract

We developed a novel compassion-focused training (mindfulness-based compassionate living; MBCL) and examined its effects in a heterogeneous psychiatric outpatient population with regard to feasibility and changes in levels of depression, anxiety, mindfulness and compassion. The training consisted of nine weekly 2.5-h sessions. Thirty-three patients, who had followed a mindfulness-based stress reduction (MBSR) program or a mindfulness-based cognitive therapy (MBCT) program beforehand, participated in the study (mean age 48.1 years; 82 % female). Participants completed self-report questionnaires before and directly after the MBCL training. Levels of depression, but not of anxiety, reduced, and levels of mindfulness and self-compassion increased. Serious limitations of this study are the small sample size, the lack of a control group and the fact that about half of the participants did not complete the posttraining questionnaires. However, we determined that it is feasible to conduct further research on this novel MBCL training program as a basis for more robust empirical investigation in the future, more specifically examining the effects of MBCL and preferably also the underlying working mechanisms.

## Introduction

Self-compassion is the capacity to be sensitive to pain and suffering of ourselves and others, accompanied by the commitment to alleviate it. Self-compassion increases with practicing mindfulness and is thought to be a mediator for stress reduction in mindfulness-based stress reduction (MBSR) and for preventing recurrence of depression in mindfulness-based cognitive therapy (MBCT) (Kuyken et al. 2010; Shapiro et al. 2005; Shapiro et al. 2007). Offering more explicit practice in self-compassion could well be of benefit to many.

It has been a consistent finding that greater self-compassion as measured by the Self-Compassion Scale (Neff 2003) correlates with less psychopathology as shown in a meta-analysis by MacBeth and Gumley (2012). Neff (2003) operationalized self-compassion by distinguishing three components: *self-kindness* as opposed to self-criticism, *common humanity* as opposed to self-isolation and *mindfulness* of painful experience as opposed to over-identification. Judging oneself harshly, experiencing oneself as isolated from supportive others and over-identifying with negative thoughts and feelings are all strongly associated with anxiety, depression and other mental health problems (Braehler et al. 2013; Neff and Dahm in press; Neff and Germer 2013). As many patients in mental health settings share these characteristics, it makes sense to design trans-diagnostic group programs for clinical settings that offer tools to cultivate self-compassion and its three components as antidotes against these unwholesome psychological tendencies. At the physiological level, compassion practice is believed to tap into evolutionary old mammalian systems designed for social bonding as it deactivates the threat system in favour of the soothing system (Gilbert and Irons 2005). Mechanisms supposed to be involved are a decrease in cortisol levels and an increase in oxytocin levels, vagal tone and heart-rate variability (Kok and Fredrickson 2010; Olff et al. 2013; Porges 2007; Rockliff et al. 2008).

There are also arguments for caution, however, as adverse reactions associated with an activated threat system can also occur when introducing self-compassion practice, particularly in those who are already highly self-critical (Longe et al. 2010; Rockliff et al. 2008). Germer (2009) called this phenomenon ‘backdraft’, and Gilbert (2010) argued how those with a background of trauma and neglect can be conditioned to respond to positive emotions with fear and therefore should be gradually introduced to compassion-focused work. The founders of MBCT expressed reservations about offering explicit practice in self-compassion to patients with recurrent depression because it could easily evoke adverse effects and feelings of failure (Segal et al. 2013). Based on their clinical view, they considered it safer to first establish basic mindfulness skills and to introduce a compassionate attitude implicitly by embodying it as a teacher. It should be mentioned that other scholars have argued that there is insufficient empirical evidence to support this view (Neff and Dahm in press). It therefore remains a hypothesis that mindfulness training should precede the safe introduction of compassion training in vulnerable patients, predominantly based on clinical arguments.

There is considerable evidence for the efficacy of mindfulness-based group interventions with groups of various psychiatric disorders studied in homogenous and mixed groups (Chiesa and Serretti 2011; Hofmann et al. 2010; Khoury et al. 2013; Klainin-Yobas et al. 2012; O'Reilly et al. 2014; Piet and Hougaard 2011; Vollestad et al. 2012; Wanden-Berghe et al. 2011; Zgierska et al. 2009).

Recently, several specific loving kindness and self-compassion training programs have been developed and studied in non-clinical samples, with promising results (Fredrickson et al. 2008; Jazaieri et al. 2013; Neff and Germer 2013; Pace et al. 2009, 2010; Wallmark et al. 2013). In these programs, no preliminary experience with mindfulness is required. In clinical settings, there is some evidence for efficacy of compassion-focused therapy (CFT) and compassionate mind training (a group program based on CFT), offered to mixed groups of patients with moderate to severe mental health problems who showed significant reductions in depression, anxiety, shame and self-criticism (Gilbert and Procter 2006; Judge et al. 2012; Lucre and Corton 2013). There is also preliminary evidence for patients with eating disorders (Gale et al. 2014), and patients with persistent psychotic phenomena (Mayhew and Gilbert 2008). Even patients in a high security ward benefited from group CFT with large effects on levels of depression and self-esteem (Laithwaite et al. 2009). Another non-controlled study of a group program offering loving kindness practice showed a significant decrease in negative symptoms and increase in positive emotions in outpatients with schizophrenia (Johnson et al. 2009). A first randomized controlled trial of group CFT for patients with schizophrenia showed that the intervention group had clinically improved more with less depressive symptoms and experience of social exclusion (Braehler et al. 2013). An early systematic review considered CFT as a promising intervention for mood disorders, particularly in those high in self-criticism (Leaviss and Uttley 2015).

The aim of this study was to examine the effects of a new mindfulness-based compassion training program, mindfulness-based compassionate living (MBCL), in a heterogeneous psychiatric outpatient population with regard to feasibility and to changes in levels of depression, anxiety, mindfulness and compassion.

## Method

### Participants

Participants were psychiatric outpatients attending MBCL training at the Center for Integrative Psychiatry (CIP; Lentis Mental Health Organization, Groningen, The Netherlands). Inclusion criteria for participating in the training were: age 18 years or older, having previously completed an MBSR or MBCT training (at the CIP or elsewhere) without adverse effects, being able to participate in a group, no physical impediments to follow the training, no alcohol or drug dependence, sufficient motivation to do home practice, being in a psychiatric condition that is judged safe enough to do further mindfulness-based work and compassion-focused exercises, having realistic expectations of the training (understanding that MBCL builds on already established mindfulness skills and that exercises in the training can be emotionally challenging; willingness to work with difficult experiences mindfully, such as re-experiencing traumatic events; knowing whom to approach in crisis situations). Fulfilment of these criteria was assessed in clinical interviews by the trainers prior to the training. If other than referring therapists were involved, their consent was required for their patients to follow the training. Involved therapists and patients had to be in agreement that the MBCL training would be followed without other simultaneous psychotherapeutic or psychopharmacological interventions other than treatment as usual (fortnightly to monthly monitoring by a nurse practitioner or psychologist; routine control by a nurse practitioner or trainee psychiatrist for those on medication; crisis intervention if needed). Participation in the study was voluntarily, as part of the standard monitoring of all treatment programs at the CIP. Our study was qualified as being exempt from review by a medical ethics committee on the basis of the regulations of the Central Committee on Research in Human Subjects Act (WMO in Dutch), given the low frequency of the assessments and the psychologically non-probing nature of the questions. Written informed consent was obtained from all individual participants included in the study.

### Procedure

At the CIP, a mindfulness-based compassion training was developed called ‘Mindfulness-Based Compassionate Living’ training (MBCL; Van den Brink and Koster 2012) for psychiatric patients who had already followed MBSR or MBCT.

The MBCL program is similar in structure to a MBSR/MBCT course, with eight thematic sessions and a silent session with guided meditations only. All exercises build on skills acquired in previous mindfulness practice, and most are guided in the group sessions. They are given as audio material and transcripts in the workbook to support home practice. An important difference with MBSR/MBCT is that in MBCL training, a range of suggestions for home practice are given following each session, rather than specific homework. This supports participants to tune into their deeper needs and to compassionately choose the exercises that connect best to their learning process. For each session, a number of exercises are added to be explored in the session and at home. Participants can always continue practising what was offered in earlier sessions or return to basic mindfulness exercises. Several elements are secular adaptations from traditional practices, such as *metta* (loving kindness meditation), where one mindfully sends kind wishes to oneself or others; *tonglen* (renamed ‘Compassionate Breathing’), where one imagines inhaling what is painful in oneself or other persons and exhaling a wholesome energy which relieves the pain; or the *Brahmaviharas* (the four immeasurables, which we call ‘Four Friends for Life’), where one practises with four self-transcending attitudes that complement each other (kindness, compassion, sympathetic joy and equanimity). Soothing breathing rhythm and compassionate imagery (safe place; compassionate companion; compassion mode) and letter writing are adapted from CFT (Gilbert 2010). Compassionately dealing with resistance, desire and forgiveness exercises were adapted from Brach (2004). Other exercises are adapted from Germer (2009), Hanson (2013), Hayes et al. (1999) and Neff (2011). Important short informal practices are the breathing space with kindness (to be practised any moment) and the breathing space with compassion (to be practised in difficult moments), extensions from the three-minute breathing space in MBCT (Segal et al. 2013). Calendar exercises are meant to help with practising mindful compassion in daily life. Dealing with the backdraft phenomenon (Germer 2009) or the fear of compassion (Gilbert 2010) is an essential part of the training. It is dealt with inside rather than outside the training and processed by giving psychoeducation on the phenomenon in order to normalise the experience, deal with it mindfully, and compassionately adjust the pace and focus of the practice, slowly exposing oneself from less to more challenging emotional content.

Patients, who were judged eligible for MBCL training by their therapist, were first informed globally about this training during a regular therapy session. Those wanting to participate were subsequently informed in detail on the procedure and on the accompanying study in a confirmation letter, together with the request to sign the informed consent form and to complete the pretraining questionnaires just before the start of the first training session at the center and bring all documents along to the first training session in a closed envelope. The trainers would subsequently hand the closed envelopes to KvdP, who was in charge of the data entry. Posttraining questionnaires were sent to the participants’ home addresses immediately after the last training session by the secretary, with the request to return them by post in the enclosed stamped envelope. In case of non-response, the secretary or KvdP attempted to remind participants by telephone in order to still obtain the questionnaires. All envelopes were opened, and data were entered into the database anonymously by KvdP, who was not involved in the training sessions. Participants were not rewarded for completing the questionnaires.

#### Training

The training consisted of nine weekly 2.5-h sessions (for details see Van den Brink and Koster 2012). Participants were offered a workbook and audio CD’s with exercises. The trainers had discussed in advance that participants should not miss more than three training sessions; otherwise, they were advised to follow the training at a later stage. An overview of the contents of the training sessions is given in Table 1.

**Table 1:**
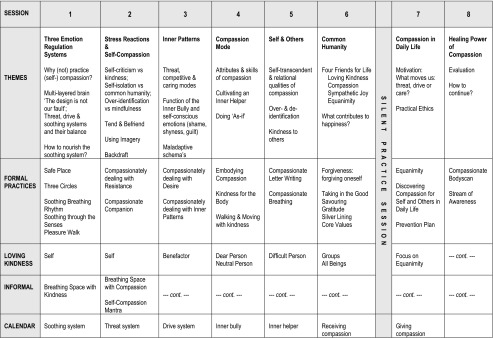
MBCL session overview

#### Trainers

Each of the training groups was led by one of the two certified mental health professionals and mindfulness teachers who developed the MBCL program themselves (Van den Brink and Koster 2012). Both are trained in MBSR and MBCT and are certified by the Dutch Association for Mindfulness-Based Trainers (VMBN). They have extensive meditation experience (20 and 35 years, respectively) and have been engaged in ongoing meditation practices, in personal as well as in professional contexts (e.g. as teachers/supervisors at the Dutch Institute for Mindfulness, the Institute for Mindfulness-Based Approaches and as leaders of retreats). One of the trainers is a psychiatrist and psychotherapist, the other a specialized psychiatric nurse and meditation teacher who has spent six years in Asia as a Buddhist monk, practicing Vipassana and loving kindness meditation and studying Buddhist psychology. Both have offered MBSR and MBCT in mental health care settings from the very first introduction of these programs in Dutch mental health care and are considered two of the Dutch pioneers in this field. They frequently teach at various mindfulness training institutions across Europe. Both trainers studied compassion-focused therapy and mindful self-compassion, and followed training seminars with founding teachers (a.o. Germer, Gilbert, Irons and Neff).

### Measures

Patients were requested to complete four frequently used valid and reliable self-report questionnaires, before and after the training.

#### Depression

Severity of depression was measured with the 21-item self-report Beck Depression Inventory-II (Beck et al. 1996; Dutch version: Van der Does 2002). Each item has to be rated on a 4-point scale, with a total score ranging from 0 to 63, higher scores denoting a higher level of depression. The internal consistency of the BDI-II in the current study was good (Cronbach’s alpha = 0.90).

#### Anxiety

The 7-item Generalized Anxiety Disorder scale (GAD-7; Spitzer et al. 2006; Dutch version: Donker et al. 2011) is an efficient self-report tool for screening for and assessing severity of generalized anxiety disorder. Each item has to be rated on a 4-point scale, with a total score ranging from 0 to 21, higher scores denoting a higher level of anxiety. In this study, the scale’s reliability was good (α = 0.87).

#### Mindfulness

The 39-item Five Facet Mindfulness Questionnaire (FFMQ; Baer et al. 2008; Dutch version: Bohlmeijer et al. 2011) assesses five aspects of mindfulness (observing, describing, acting with awareness, non-judging of inner experience and non-reactivity to inner experience). Each item has to be rated on a 5-point scale (ranging from 1 to 5), higher scores denoting a higher level of mindfulness. Here, we only used the FFMQ total score. Cronbach’s alpha in the current study was adequate (α = 0.87).

#### Self-Compassion

The 24-item Dutch version (Neff and Vonk 2009) of the Self-Compassion Scale (SCS; Neff 2003) measures the degree of self-compassion. The SCS has six subscales (self-kindness, self-judgement, common humanity, isolation, mindfulness and over-identification). Each item has to be rated on a 5-point scale (ranging from 1 to 5), higher scores denoting a higher level of self-compassion. Here, we only used the SCS total score. Reliability of the SCS in this study was good (α = 0.92).

#### Diagnosis

The DSM-IV diagnosis at baseline was set by patient’s main therapist in a clinical interview (DSM-IV-TR; American Psychiatric Association 2000). All main therapists were experienced psychiatrists and psychologists and well trained in applying DSM-IV-TR.

#### Demographics

At the pretraining assessment, patients filled in their gender, birth date and how often they had done mindfulness exercises in the past month. Data on educational level, living situation, illness duration, diagnostic category and comorbid Axis-II disorders were collected from their medical records.

### Data Analyses

Analyses were carried out with the statistical package IBM SPSS, version 23. All continuous variables were tested for normality with the Kolmogorov-Wilk test, which has to be used in relatively small (*n* < 50) samples. Non-normally distributed variables were log-transformed for performing *t* tests and regression analyses. This was the case for the variables ‘age at assessment’, ‘illness duration’, and ‘FFMQ (total)’. Diagnoses were categorized as follows: Axis-I: mood, anxiety or other disorder; Axis-II: personality disorder, deferred or no diagnosis). Educational level and living situation were dichotomized (high/low respectively living alone/together).

To analyse whether there were differences between completers and non-completers (i.e. participants who did not fill in the posttraining questionnaires, but did attend all MBCL sessions) in gender, age, educational level, living situation, illness duration, diagnostic category, comorbid Axis-II disorders and total scores on BDI-II, GAD, FFMQ and SCS at pretraining assessment, we used independent *t* tests for continuous variables and chi-square tests for categorical variables. Differences between pre and post MBCL training scores were examined with paired-samples *t* tests, and Cohen’s *d* was calculated as a measure of the effect size for paired-samples *t* tests by dividing the mean difference by its standard deviation, with a *d* value between 0 and 0.3 being considered a small effect size, between 0.3 and 0.6 a moderate effect size, and over 0.6 a large effect size).

## Results

All 62 patients attended the minimum required number of MBCL training sessions (i.e. at least six of nine sessions). Thirty-three patients (53 % of 62 patients) completed the questionnaires before and after MBCL. Their mean age was 48.1 years (SD = 12.8; range 23.7–65.9), 82 % were female, 70 % had a high educational level and 42 % were living alone. Mean illness duration was 13.1 years (SD = 11.4; range 0.7–42.7). Diagnostic categories were mood (*n* = 21; 64 %), anxiety (*n* = 8; 24 %) and other (*n* = 4; 12 %) disorders; 48.5 % of the sample were diagnosed with Axis-II comorbidity. There were no significant differences between completers and non-completers in gender, age, educational level, living situation, illness duration, diagnostic category, comorbid Axis-II disorders and total scores on BDI-II, GAD, FFMQ and SCS at pretraining measurement (see Tables 2 and 3).

**Table 2:** Characteristics of completers and non-completers analysed with Chi-square or *t* tests (*N* = 62)

	Completers (*n* = 33)		Non-completers (*n* = 29)		Differences	
	Mean (range)	SD	Mean (range)	SD	*χ* ^2^ (df1) or *t* (df60)	*p*
Gender (% female)	81.8		79.3		0.06	0.80
Age^a^	48.1 (23.7–65.9)	12.8	47.1 (24.2–69.3)	13.0	−0.28	0.78
Educational level (% high)	69.7		53.6		1.68	0.20
Living situation (% living alone)	42.4		62.1		2.39	0.12
Illness duration^a^	13.1 (0.7–42.7)	11.4	9.0 (1.0–21.7)	6.1	−1.22	0.23
Diagnostic category (%)					5.77	0.06
Anxiety (Axis I)	24.2		20.7			
Depression (Axis I)	63.6		41.4			
Other (Axis I)	12.1		37.9			
Axis-II comorbidity (%)^b^	48.5		51.7		2.26	0.32

*SD* standard deviation, *MF* mindfulness

^a^Differences were calculated with the log-transformed scores

^b^df = 2

**Table 3:** Mean differences in pre MBCL training scores between completers and non-completers analysed with *t* tests (*N* = 62)

	Pre MBCL training scores		Pre MBCL training scores		Differences	
	Non-completers		Completers			
Outcome	Mean (range)	SD	Mean (range)	SD	*t* (df60)	*p*
BDI-II (total)	21.4 (4.0–40.0)	9.7	20.1 (1.0–46.0)	10.9	0.46	0.648
GAD (total)	9.0 (1.0–18.0)	4.0	7.8 (0.0–17.0)	4.8	1.10	0.276
FFMQ (total means)^a^	2.91 (2.15–4.00)	0.38	3.07 (2.31–4.24)	0.41	−1.60	0.116
SCS (total means)	2.50 (1.58–3.83)	0.54	2.67 (1.58–4.33)	0.65	−1.09	0.280

Means and standard deviations reported on unstandardized scores

*MBCL* mindfulness-based compassionate living training, *SD* standard deviation, *BDI-II* Beck Depression Inventory-II, *GAD* Generalized Anxiety Disorder scale, *FFMQ* Five Facet Mindfulness Questionnaire, *SCS* Self-Compassion Scale

^a^Differences were calculated with the log-transformed scores

In the month before the MBCL training, 22 % of the patients had done mindfulness exercises at least five times a week, 25 % 3 or 4 times a week, 28 % 1 or 2 times a week and 16 % 1 or 2 times that month, whereas 9 % had not practised.

This study showed that the training was applicable in a heterogeneous patient sample, mainly with anxiety and depression. All patients completed the training. Crisis situations or adverse incidents related to the training were not encountered by the trainers. From participants themselves or their therapists came no reports of concern regarding the training, other than the expected backdraft phenomenon. This was frequently encountered according to the trainers and could be worked with constructively during sessions. Dealing with backdraft indeed proved an important part of the training, generating more insight and self-compassion. Occasionally, participants asked for an extra individual consultation with their trainer or mental health practitioner which helped them to continue with the program. This was considered normal practice within this group of patients following group trainings, as it is common during other types of training as well.

Table 4 shows the mean pre and posttraining scores (SD) for all the study variables. After the MBCL training, level of depression decreased 25.4 %, level of mindfulness increased 6.8 % and level of self-compassion increased 13.1 %. The effect size for the level of compassion was large, and the effect sizes for depression and mindfulness were moderate. We found no significant changes in anxiety.

**Table 4:** Pre and post MBCL training test scores and MBCL effects analysed with paired-samples *t*-tests and Cohen’s *d* effect size (*N* = 33)

	Pre MBCL training scores		Post MBCL training scores		Differences		Effect size
Outcome	Mean (range)	SD	Mean (range)	SD	*t* (df32)	*p*	Cohen’s *d*
BDI-II (total)	20.1 (1.0–46.0)	10.9	15.0 (0.0–44.0)	10.1	3.38	0.002*	0.59
GAD (total)	7.8 (0.0–17.0)	4.8	7.0 (0.0–18.0)	4.9	1.02	0.316	0.18
FFMQ (total means)^a^	3.07 (2.31–4.24)	0.41	3.28 (2.36–4.15)	0.46	−3.02	0.005*	0.53
SCS (total means)	2.67 (1.58–4.33)	0.65	3.02 (1.83–3.96)	0.58	−3.86	0.001*	0.67

Means and standard deviations reported on unstandardized scores

*MBCL* mindfulness-based compassionate living training, *SD* standard deviation, *BDI-II* Beck Depression Inventory-II, *GAD* Generalized Anxiety Disorder scale, *FFMQ* Five Facet Mindfulness Questionnaire, *SCS* Self-Compassion Scale

^a^Differences were calculated with the log-transformed scores

*p < 0.01

## Discussion

A novel compassion-focused training program, the ‘Mindfulness-Based Compassionate Living’ training (MBCL), was examined in this feasibility study.

An important difference between MBCL and mindful self-compassion (MSC; Neff and Germer 2013) and compassion-focused therapy (CFT; Gilbert 2010) is that MBCL builds on a prerequisite mindfulness training. MBCL was developed in a clinical setting, contrary to the MSC program by Neff and Germer (2013), and has roots in the tradition of mindfulness-based cognitive therapy and mindfulness-based stress reduction with regard to the way the program is offered. MSC introduces mindfulness practice in the program itself. Both MSC and MBCL include loving kindness meditation as a way to foster a kind attitude towards oneself and others, and formal and informal self-compassion practices when one meets pain and suffering.

Another difference with MSC is that MBCL integrates much of the educational material (such as the evolutionary view and the emotion regulation systems) and practices used in CFT (Gilbert 2010, 2014), which can be particularly helpful in clinical settings (Leaviss and Uttley 2015). MBCL differs from CFT in that it is a mindfulness-based group training rather than a group therapy, which means that participants deepen the work of self-inquiry started in MBSR/MBCT, guided by a mindfulness teacher who delivers the course not in the role of a therapist, but of a trainer who teaches skills how to help oneself.

The rationale for the MBCL program is that in a clinical setting, explicit practice in self-compassion could well be more beneficial and safer when offered to those who already had the experience of a mindfulness course and time to develop basic mindfulness skills, taking into account that for many patients with chronic or recurrent mental health problems an eight-week course is not a substantial amount of time. Many need follow-up sessions, follow a second or third mindfulness course and encounter great difficulty when invited for more explicit practice in self-kindness and compassion. Once they learn to be with their experience as it is, and be gentle and kind whilst experiencing mild discomfort, like they do in basic mindfulness training, they are better prepared to stay with more painful experiences and meet these with compassion.

The training appeared to be feasible and safe, in line with mindfulness meditation-based interventions (Zgierska 2009), as no training-related incidents were reported during or between the training sessions. With respect to this, the following two factors can have played a role in this. First, only those with prior experience with mindfulness training could participate. Second, only participants with psychiatric problems that were considered to be safe with respect to participation in our self-compassion based intervention were allowed to participate.

After the MBCL training, level of depression had decreased (with a moderate effect size), and levels of mindfulness and self-compassion had increased (with a moderate and large effect size, respectively). The mean pretraining BDI-II score is comparable to that reported for psychiatric outpatients in general (Beck et al. 1996), but lower than in other studies in outpatients with a major depressive disorder (see Quilty et al. 2010 and Steer et al. 1999). The mean pretraining GAD-7 score is higher compared to primary care patients and to the general population, but lower than in patients with an anxiety disorder (Loewe et al. 2008). Level of anxiety had decreased after the training, but this result did not reach statistical significance. As we did not have a follow-up assessment, we cannot make any statements about whether improvements were maintained in completers. A study on MBCT by Ruths and colleagues (2013) found additional improvements in trait anxiety in participants who continued MBCT practice at 3-month follow-up.

Because self-compassion—like mindfulness (Bos et al. 2014)—is likely to be beneficial to clients with various forms of psychopathology (MacBeth and Gumley 2012), it has logistic and possibly therapeutic advantages to offer MBCL as a transdiagnostic training program to heterogeneous groups. Obviously, further research is needed to support this.

### Limitations and Strengths

The results of this feasibility study need to be interpreted with considerable caution, given the absence of a control group. Therefore, we cannot draw firm conclusions about the extent to which the observed improvements are due to the intervention people received. Other important limitations are the low response rate and the small sample size. Assuming a 5 % two-sided type I error rate and an 80 % power, the given sample size of 33 persons can detect medium and high effect sizes (Cohen’s *d* = 0.5–0.8), whereas small effects may not reach the level of significance. With respect to the response rate, almost half of the participants did not complete the posttraining questionnaires. This is in line with earlier findings of using routine outcome measurements, as we did for the current pilot study, where response rates were even lower (Hoenders et al. 2014). Although we did not observe important differences between the completers and the non-completers with respect to participants’ characteristics, nor to pretraining scores, this low response rate may have induced selection bias and may affect the generalization of the results. In order to avoid non-response, the pre- and posttraining assessments are now part of the first and the last MBCL session at our center. Participants will hand the completed questionnaires in closed envelopes to the trainers, who hand them to the researchers who are not involved in the MBCL training, for anonymous data entry.

Furthermore, the absence of any formal and/or external checks on program fidelity should be mentioned as a limitation. Also, investigator allegiance can be considered as a limitation, although except from EvdB and FK who offered the training, the other researchers were independent.

Importantly, although the trainers did not notice crisis situations or adverse incidents related to the training, a limitation in this feasibility study is that safety and/or adverse effects should have independently been monitored by an ethical review board or a trial steering committee. Furthermore, the majority of the sample were female. In our study, women had lower self-compassion scores than men. This is in line with the results of a recent meta-analysis of gender differences in self-compassion (Yarnell et al. 2015) reporting that women had slightly lower self-compassion scores than men, similar to the study of Mercadillo (2011). This may explain the high percentage of female participants. Finally, this study lacks objective observational measures such as social or vocational functioning or relapse rates in order to validate the change in depression and anxiety clinically.

Despite all aforementioned limitations, we determined that it is feasible to conduct further research on this novel MBCL training program as a basis for more robust empirical investigation in the future, including a randomized controlled trial, more specifically examining the effects of MBCL and preferably also the underlying working mechanisms (see also Kazdin 2007). Also, possible evidence for differential outcome in participants from differential diagnostic groups should be examined in larger samples.
